# Supplementary material for: Association of relative handgrip strength on the development of diabetes mellitus in elderly Koreans
Source: PLoS One. 2024 Oct 31;19(10):e0309558. doi: 10.1371/journal.pone.0309558 (PMC11527183; doi:10.1371/journal.pone.0309558)
Supplement: Attachment — Submitted filename: Recommendations.docx [file pone.0309558.s004.docx]

Manuscript Title: Association of relative handgrip strength on the development of diabetes mellitus in elderly Koreans.

The study investigates the correlation between relative handgrip strength (HGS) and the likelihood of developing diabetes in a Korean population cohort of 22,016 older adults. The research presents valuable insights into the potential relationship between relative HGS and diabetes risk, although several methodological and interpretational aspects require further consideration.

**Major comments:**

- The authors should compare the predictive abilities of relative HGS, absolute HGS, and BMI in predicting diabetes development to ascertain any additional value provided by relative HGS over other measurements. Furthermore, it would be beneficial to assess whether relative HGS augments the predictive capacity of measured levels of physical activity in predicting new-onset diabetes among older adults.
- The study limitations acknowledge using self-reported height and weight variables, which may introduce recall bias, particularly among older adults. Given the centrality of these variables to the calculation of relative HGS, the authors should explore alternative methods, such as using measured height and weight or employing absolute HGS. Additionally, utilizing self-reporting to diagnose new-onset diabetes may introduce unreliability; therefore, including cases with documented medical records or employing more reliable alternatives is advisable. Incorporating only cases with reliable records, although potentially reducing the sample size, would improve the quality and reliability of the evidence obtained.
- Clarification is needed regarding how medical comorbidities were diagnosed, as this could impact the interpretation of the study findings.
- Details regarding the dynamometer used for measuring HGS should be provided to ensure transparency and replicability of the study methodology.
- The authors should mention whether cases with painful hand diseases or neurological disorders that could affect HGS were excluded from the analysis, as their inclusion could confound the results.
- Considering the complexity of the Generalized Estimating Equation (GEE) model covariance structure analysis, engaging an expert statistical reviewer to validate the approach and interpretation would strengthen the study's methodological rigor.

**Minor Comments:**

- The introduction appears lengthy and contains redundancy. Streamlining the introduction by eliminating redundant information would enhance its clarity and conciseness.
- The definition of relative HGS should be removed from the introduction and added to the methods section.

Overall, the study presents a valuable contribution to exploring the correlation between relative HGS and diabetes risk. Addressing the aforementioned comments would enhance the methodological robustness of the research findings.
