# Supplementary material for: Association of relative handgrip strength on the development of diabetes mellitus in elderly Koreans
Source: PLoS One. 2024 Oct 31;19(10):e0309558. doi: 10.1371/journal.pone.0309558 (PMC11527183; doi:10.1371/journal.pone.0309558)
Supplement: Attachment — Submitted filename: 2. Response to Reviewers_Sohn Yeo Ju.docx [file pone.0309558.s005.docx]

**# Response to Reviewers**

May/24/2024

Dear Editor:

We respectfully resubmit the revised manuscript entitled: “ Association of relative handgrip strength on the development of diabetes mellitus in elderly Koreans.” The paper was coauthored by Yeo Ju Sohn, Hong Soo Lee, Hasuk Bae, Hee Cheol Kang, Hyejin Chun, Insun Ryou, Eun Jee Chang, Sungchan Kang, Sang Wha Lee, Kyung Won Shim.

The authors greatly appreciated the careful and thoughtful review from reviewers. We are very pleased to have the opportunity to edit our manuscript. We have read the comments from the editors and reviewers carefully and have revised our paper thoroughly based on these comments. Point-by-point responses to the criticisms and suggestions are described below, and the modified contents are presented in highlight in the revised version (Marked-up Manuscript file).

In this study, we explored the relationship between relative handgrip strength (HGS) and diabetes mellitus(DM) in an elderly Korean population to recognize the importance of simultaneously evaluating the risks of low muscle strength and increased body mass index. We believe that our study significantly contributes to the literature because our findings suggest that maintaining or improving HGS through interventions such as abstinence and resistance exercises may be an effective strategy for DM prevention, particularly in older adults.

Further, we believe that this paper will be of interest to the readership of your journal because we show that relative HGS can be a useful predictive factor of DM incidence and can be utilized as a readily measurable surrogate for physical function and muscle strength in predicting DM in clinical settings.

This manuscript has not been published or presented elsewhere in part or entirety and is not under consideration by another journal. All study participants provided informed consent, and the appropriate ethics review board approved the study design. We have read and understood your journal’s policies and believe that neither the manuscript nor the study violates these. There are no conflicts of interest to declare.

We have read and understood your journal’s policies, and we believe that neither the manuscript nor the study violates any of these. There are no conflicts of interest to declare.

We kindly ask you to review the amendments, and we eagerly anticipate your favorable consideration in the future. Thank you for your consideration. I look forward to hearing from you.

Sincerely,

Yeo Ju Sohn

Department of Family Medicine, Ewha Womans University Seoul Hospital, Ewha Womans University College of Medicine, Gonghang-daero 260, Gangseo-gu, Seoul, Korea

Tel: +82-10-2286-2579, Fax: +82-2-2654-2439 E-mail: yeoju822@naver.com

ORCID : https://orcid.org/0000-0002-1196-4011

**Reviewer #1:** This manuscript explore the role of relative handgrip strength for predicting the incidence of diabetes diagnosis in the elder population. It raised the interest of readers about the significance of handgrip strength on preventing diabetes in the elderly. However, several questions need to be elucidated clearly.

First, please specify the BMI categories in the methodology with adequate reference citation. The ordinary terms of underweight, normal, overweight, and obesity were suggested, rather than thin, moderate, overweight, and obesity. And the criteria for Asian population must have different interpreting cut off value by WHO.

We sincerely appreciate the valuable feedback provided by the reviewer. Based on their insightful comments, we have made significant revisions to our study. We have revised the Introduction as follows.

We defined cutoffs for underweight (<18.5 kg/m2), normal (18.5–22.9 kg/m2), overweight (23.0–24.9 kg/m2), and obesity(≥25.0 kg/m2) based on the WHO Asia-Pacific regional guidelines.[1]

Second, BMI had a main impact on the calculated relative HGS. For example, those obese population would mostly have lower relative HGS after calculation, while obesity is the critical predictor for diabetes incidence. There should be a clear rationale for relative HGS, and it was suggested to have a comparison with absolute HGS, or HGS adjusted for height square. Current calculation of relative HGS may have a main concern of collinearity with BMI. And please have a discussion on the relationship between relative HGS and BMI in depth.

We sincerely appreciate the valuable feedback provided by the reviewer. Based on their insightful comments, we have made significant revisions to our study. We have revised the Introduction as follows.

HGS is an indicator with a significant correlation with sarcopenia, which refers to muscle loss with age, and serves as a basic indicator to evaluate overall physical ability and muscle function, especially in the elderly population. According to previous studies, it has been revealed that there is a correlation between BMI and HGS. [2] [3] Additionally, it has been reported that there is a deep correlation between a decrease in muscle mass and a decrease in HGS grip strength. [4] Previous studies have demonstrated the usefulness of absolute handgrip strength in the identification of various health problems and its potential as a new vital sign across the lifespan, but have shown conflicting results. [5] The confounding effect of body size was thought to be one of the causes. In other words, previous studies conducted only with the concept of absolute HGS showed inconsistent results, and this was the result of using absolute HGS as an indicator of muscle strength without body mass correction, as absolute grip strength is closely related to body mass index. Relative grip strength has therefore been recommended as a better metric to take into account the effects of both body mass and muscle strength, and this measure helps account for differences in hand grip strength that may be influenced by an individual's overall size. Various previous studies have demonstrated that relative HGS may be beneficial in predicting cardiovascular biomarkers, metabolic profile, and risk of other cardiometabolic disorders. [6] [7] [8] In addition, there are previous research results showing that relative HGS can predict new-onset DM better than absolute HGS. A study conducted in middle-aged and older adults in Europe also found that low HGS was an independent predictor of new-onset diabetes risk, suggesting that relative HGS had a slightly higher predictive ability for future diabetes than absolute HGS in people aged 50 years or older. have emphasized that screening for low HGS may be of value in preventing diabetes.[7]

We sincerely appreciate the valuable feedback provided by the reviewer. Based on their insightful comments, we have made significant revisions to our study. We have revised the discussion as follows.

Obesity, which corresponds to a high BMI, is an important predictor of developing diabetes mellitus, but there also exist metabolically obese normal weight phenotype or Obesity paradox. Some people have a high BMI but still have a high muscle mass, and although obesity increases various cardiovascular disease risk factors, patients with various types of CVD sometimes have a better prognosis if they are classified as overweight or obese. This phenomenon may be viewed as being due to the “low-fat paradox,” as individuals classified as normal or underweight have a poor prognosis for cardiovascular disease. [9] There is also visceral fat type obesity with a low BMI but high visceral fat, or obesity with only a high waist circumference, and it is difficult to sufficiently predict diabetes using the BMI indicator alone. In contrast to lower body weight, high muscle strength levels or high HGS in old age are known to play a protective role against the risk of premature death or cardiometabolic disease such as diabetes mellitus. According to a recent study, it has been reported that grip strength, which represents upper body strength, is more closely related to mortality or metabolic diseases including Type 2 Diabetes Mellitus than lower body strength. [10] In a study on leaner Japanese Americans, it was found that in people with a BMI in the bottom 25%, the greater the grip strength, the lower the risk of developing diabetes mellitus. [11] There are cases where the BMI is high but the muscle strength is high due to hard exercise, and it is difficult for absolute HGS to consider these cases. Therefore, in this study, relative grip strength was defined as an independent variable to determine whether the risk of diabetes mellitus is high when low muscle strength and high BMI coexist.

Third, it was suggested to present BMI distribution in both table 3 and 5. There would be a strong interaction between relative HGS and BMI, while BMI might play a more critical role on predicting diabetes incidence. Therefore, it was suggested to include BMI for analysis, and to create a interaction term between BMI and relative HGS in GEE model. Otherwise, there should be a clear rationale why BMI was not included.

We sincerely appreciate the valuable feedback provided by the reviewer. Based on their insightful comments, we have made significant revisions to our study. We have revised Tables 3 and 5 and Results according to the reviewer’s recommendations. As suggested, we have incorporated BMI into our analysis. Specifically, we created an interaction term between BMI and relative handgrip strength (HGS) in the generalized estimating equation (GEE) model. Concerns about collinearity between BMI and relative HGS were noted, as this relationship led to increased p-values. However, it is noteworthy that despite this collinearity, a consistent trend remains: higher handgrip strength is associated with a reduced odds ratio (OR) for newly-onset diabetes mellitus (DM) in both men and women. Therefore, while acknowledging the collinearity issue, we believe that relative HGS serves as a novel surrogate marker for predicting DM risk and contributes to our understanding of preventive strategies for older adults. Once again, we express our gratitude for the constructive feedback, which has undoubtedly strengthened the scientific rigor of our study. We remain committed to advancing our understanding of DM risk factors and preventive measures.

Final, some minor errors were suggested to check again. Such as the flow chart for participants inclusion, 63125 minus 29514 leaves 33611, not 33701. And the number should be presented as mean +/- SD in each tertile group in table 3.

We sincerely appreciate the valuable feedback. It seems there was an error in the calculation process. For those under 65 years of age, the correct number is not 29,514 but 29,424. We have made the necessary correction.

Reviewer #2: Manuscript Title: Association of relative handgrip strength on the development of diabetes mellitus in elderly Koreans.

The study investigates the correlation between relative handgrip strength (HGS) and the likelihood of developing diabetes in a Korean population cohort of 22,016 older adults. The research presents valuable insights into the potential relationship between relative HGS and diabetes risk, although several methodological and interpretational aspects require further consideration.

Major comments:

• The authors should compare the predictive abilities of relative HGS, absolute HGS, and BMI in predicting diabetes development to ascertain any additional value provided by relative HGS over other measurements. Furthermore, it would be beneficial to assess whether relative HGS augments the predictive capacity of measured levels of physical activity in predicting new-onset diabetes among older adults.

We sincerely appreciate the valuable feedback provided by the reviewer. Based on their insightful comments, we have made significant revisions to our study. We have revised the introduction part as follows.

Previous studies have demonstrated the usefulness of absolute handgrip strength in the identification of various health problems and its potential as a new vital sign across the lifespan, but have shown conflicting results. [5] The confounding effect of body size was thought to be one of the causes. In other words, previous studies conducted only with the concept of absolute HGS showed inconsistent results, and this was the result of using absolute HGS as an indicator of muscle strength without body mass correction, as absolute grip strength is closely related to body mass index. Relative grip strength has therefore been recommended as a better metric to take into account the effects of both body mass and muscle strength, and this measure helps account for differences in hand grip strength that may be influenced by an individual's overall size. Various previous studies have demonstrated that relative HGS may be beneficial in predicting cardiovascular biomarkers, metabolic profile, and risk of other cardiometabolic disorders. [6] [7] [8]

And, after analysis including the effect of BMI, revised versions of tables 3 and 5 of the main text were included in the revised manuscript. We have also revised the discussion part in the revised manuscript.

• The study limitations acknowledge using self-reported height and weight variables, which may introduce recall bias, particularly among older adults. Given the centrality of these variables to the calculation of relative HGS, the authors should explore alternative methods, such as using measured height and weight or employing absolute HGS. Additionally, utilizing self-reporting to diagnose new-onset diabetes may introduce unreliability; therefore, including cases with documented medical records or employing more reliable alternatives is advisable. Incorporating only cases with reliable records, although potentially reducing the sample size, would improve the quality and reliability of the evidence obtained.

We sincerely appreciate the valuable feedback provided by the reviewer. Based on their insightful comments, we have made significant revisions to our study. We have revised the limitation part in the revised manuscript.

• Clarification is needed regarding how medical comorbidities were diagnosed, as this could impact the interpretation of the study findings.

We sincerely appreciate the valuable feedback provided by the reviewer. Based on their insightful comments, we have made significant revisions to our study. We have revised the methods part in the revised manuscript.

• Details regarding the dynamometer used for measuring HGS should be provided to ensure transparency and replicability of the study methodology.

• The authors should mention whether cases with painful hand diseases or neurological disorders that could affect HGS were excluded from the analysis, as their inclusion could confound the results.

We sincerely appreciate the valuable feedback provided by the reviewer. Based on your insightful comments, we have made significant revisions to our study. We have revised the Materials and methods part.

The HGS gauge used in the KLoSA was the Tanita 6103 model. HGS measurement was conducted with the principle of identifying whether the respondent is in a state where measurement is possible, and measurement is not conducted if the respondent does not want to or if one hand is currently injured or in pain. After confirming the possibility of HGS measurement, the HGS of the primarily used hand was measured first, followed by the measurement of the HGS of the other hand.

• Considering the complexity of the Generalized Estimating Equation (GEE) model covariance structure analysis, engaging an expert statistical reviewer to validate the approach and interpretation would strengthen the study's methodological rigor. •

Seungchan Kang's undergraduate major is statistics, and he is currently pursuing a doctoral degree at Seoul National University's Graduate School of Public Health. He is good at analyzing data in the health and medical field using Stata, R, and SAS. The data analysis of this study was conducted through Kang Seung-chan's GEE model analysis.

Minor Comments:

• The introduction appears lengthy and contains redundancy. Streamlining the introduction by eliminating redundant information would enhance its clarity and conciseness.

We sincerely appreciate the valuable feedback provided by the reviewer. Based on your insightful comments, we have made significant revisions to our study. We have revised the Introduction to streamline it.

• The definition of relative HGS should be removed from the introduction and added to the methods section.

We sincerely appreciate the valuable feedback. The definition of relative handgrip strength was removed from the introduction and added to the methods section.

Overall, the study presents a valuable contribution to exploring the correlation between relative HGS and diabetes risk. Addressing the aforementioned comments would enhance the methodological robustness of the research findings.

**References**

1.	World Health Organization. Regional Office for the Western Pacific. The Asia-Pacific perspective : redefining obesity and its treatment. Sydney : Health Communications Australia; 2000. Available: http://iris.wpro.who.int/handle/10665.1/5379

2.	Liao K-H. Hand Grip Strength in Low, Medium, and High Body Mass Index Males and Females. Middle East J Rehabil Health. 2016;3. doi:10.17795/mejrh-33860

3.	R D, C V, S M, R S. Comparative study of body mass index, hand grip strength, and handgrip endurance in healthy individuals. Natl J Physiol Pharm Pharmacol. 2017; 1. doi:10.5455/njppp.2017.7.1030007022017

4.	Chan J, Lu Y-C, Yao MM-S, Kosik RO. Correlation between hand grip strength and regional muscle mass in older Asian adults: an observational study. BMC Geriatr. 2022;22: 206. doi:10.1186/s12877-022-02898-8

5.	Vaishya R, Misra A, Vaish A, Ursino N, D’Ambrosi R. Hand grip strength as a proposed new vital sign of health: a narrative review of evidences. J Health Popul Nutr. 2024;43: 7. doi:10.1186/s41043-024-00500-y

6.	Li D, Guo G, Xia L, Yang X, Zhang B, Liu F, et al. Relative Handgrip Strength Is Inversely Associated with Metabolic Profile and Metabolic Disease in the General Population in China. Front Physiol. 2018;9: 59. doi:10.3389/fphys.2018.00059

7.	Li G, Qiao Y, Lu Y, Liu S, Ding Y, Chen X, et al. Role of handgrip strength in predicting new-onset diabetes: findings from the survey of health, ageing and retirement in Europe. BMC Geriatr. 2021;21: 445. doi:10.1186/s12877-021-02382-9

8.	Lee W-J, Peng L-N, Chiou S-T, Chen L-K. Relative Handgrip Strength Is a Simple Indicator of Cardiometabolic Risk among Middle-Aged and Older People: A Nationwide Population-Based Study in Taiwan. PLoS One. 2016;11: e0160876. doi:10.1371/journal.pone.0160876

9.	Elagizi A, Kachur S, Lavie CJ, Carbone S, Pandey A, Ortega FB, et al. An Overview and Update on Obesity and the Obesity Paradox in Cardiovascular Diseases. Progress in Cardiovascular Diseases. 2018;61: 142–150. doi:10.1016/j.pcad.2018.07.003

10.	Underland LJ, Schnatz PF, Wild RA, Saquib N, Shadyab AH, Allison M, et al. The impact of weight change and measures of physical functioning on mortality. J American Geriatrics Society. 2022;70: 1228–1235. doi:10.1111/jgs.17626

11.	Wander PL, Boyko EJ, Leonetti DL, McNeely MJ, Kahn SE, Fujimoto WY. Greater hand-grip strength predicts a lower risk of developing type 2 diabetes over 10 years in leaner Japanese Americans. Diabetes Research and Clinical Practice. 2011;92: 261–264. doi:10.1016/j.diabres.2011.01.007
