# Supplementary material for: Association of relative handgrip strength on the development of diabetes mellitus in elderly Koreans
Source: PLoS One. 2024 Oct 31;19(10):e0309558. doi: 10.1371/journal.pone.0309558 (PMC11527183; doi:10.1371/journal.pone.0309558)
Supplement: Attachment — Submitted filename: 2. Response to Reviewers_Sohn Yeo Ju.docx [file pone.0309558.s006.docx]

**# Response to Reviewers**

July/28/2024

Dear Editor:

We respectfully resubmit the revised manuscript entitled: “ Association of relative handgrip strength on the development of diabetes mellitus in elderly Koreans.” The paper was coauthored by Yeo Ju Sohn, Hong Soo Lee, Hasuk Bae, Hee Cheol Kang, Hyejin Chun, Insun Ryou, Eun Jee Chang, Sungchan Kang, Sang Wha Lee, Kyung Won Shim.

The authors greatly appreciated the careful and thoughtful review from reviewers. We are very pleased to have the opportunity to edit our manuscript. We have read the comments from the editors and reviewers carefully and have revised our paper thoroughly based on these comments. Point-by-point responses to the criticisms and suggestions are described below, and the modified contents are presented in highlight in the revised version (Marked-up Manuscript file).

In this study, we explored the relationship between relative handgrip strength (HGS) and diabetes mellitus(DM) in an elderly Korean population to recognize the importance of simultaneously evaluating the risks of low muscle strength and increased body mass index. We believe that our study significantly contributes to the literature because our findings suggest that maintaining or improving HGS through interventions such as abstinence and resistance exercises may be an effective strategy for DM prevention, particularly in older adults.

Further, we believe that this paper will be of interest to the readership of your journal because we show that relative HGS can be a useful predictive factor of DM incidence and can be utilized as a readily measurable surrogate for physical function and muscle strength in predicting DM in clinical settings.

This manuscript has not been published or presented elsewhere in part or entirety and is not under consideration by another journal. All study participants provided informed consent, and the appropriate ethics review board approved the study design. We have read and understood your journal’s policies and believe that neither the manuscript nor the study violates these. There are no conflicts of interest to declare.

We have read and understood your journal’s policies, and we believe that neither the manuscript nor the study violates any of these. There are no conflicts of interest to declare.

We kindly ask you to review the amendments, and we eagerly anticipate your favorable consideration in the future. Thank you for your consideration. I look forward to hearing from you.

Sincerely,

Yeo Ju Sohn

Department of Family Medicine, Ewha Womans University Seoul Hospital, Ewha Womans University College of Medicine, Gonghang-daero 260, Gangseo-gu, Seoul, Korea

Tel: +82-10-2286-2579, Fax: +82-2-2654-2439 E-mail: yeoju822@naver.com

ORCID : https://orcid.org/0000-0002-1196-4011

Dear Reviewer,

We appreciate your valuable feedback on our manuscript. Below, we address each of your comments and describe the revisions made:

Comment 1:
The revised manuscript included BMI and relative HGS for statistical analysis, while the conclusion should be very different and would be a negative study. According to the GEE results in Table 5, the relative HGS did not achieve statistical significance, while overweight and obesity did. This is a negative study, and BMI accounts for new diabetes mellitus incidence in the majority. Please review if the manuscript should be reorganized again.

Response:
We have reorganized the manuscript to reflect the significance of BMI in predicting DM incidence. The conclusion now acknowledges that relative HGS did not achieve statistical significance, and we discuss the implications of these negative findings. The manuscript has been updated to emphasize the importance of BMI and its role in the development of DM.

Comment 2:
Even BMI variable was included for analysis in GEE, it was wondered why the author did not put in trend analysis for adjustment. The table 6 was the same as the original submission. When it was disclosed BMI showed great significance in GEE, it was very reasonable to put this variable for adjustment in this study.

Response:
We have conducted a trend analysis for adjustment of BMI and included these findings in the revised manuscript. Table 6 has been updated accordingly, and the results and discussion sections now address the adjusted analysis, emphasizing the significant impact of BMI on DM incidence.

Comment 3:
It was suggested to review and recheck the organization of the content of the manuscript and to consider resubmission as a negative study.

Response:
The manuscript has been thoroughly reviewed and reorganized to present the findings as a negative study. We discuss the implications of BMI's significance and the lack of statistical significance for relative HGS. The content has been adjusted to provide a clear and accurate representation of our study's results.

We hope these revisions meet your expectations and improve the quality of our manuscript.

Sincerely,
[Yeo Ju Sohn]
